# Supplementary material for: The Specific Molecular Changes Induced by Diabetic Conditions in Valvular Endothelial Cells and upon Their Interactions with Monocytes Contribute to Endothelial Dysfunction
Source: Int J Mol Sci. 2024 Mar 6;25(5):3048. doi: 10.3390/ijms25053048 (PMC10932031; doi:10.3390/ijms25053048)
Supplement: Supplementary file 1 [file ijms-25-03048-s001.zip › ijms-2875720-supplementary.pdf]

Supplementary table S1: Data quality summary.

sample	library	raw_reads	raw_bases	clean_reads	clean_bases	error_rate	Q20	Q30	GC_pct
VEC_C1 (NG)	ERRA220045756-1a	41533514	6.23G	41349446	6.2G	0.03	97.31	92.51	49.68
VEC_C2 (NG)	ERRA220045764-1a	38059198	5.71G	37690706	5.65G	0.03	96.49	91.14	49.68
VEC_C3 (NG)	ERRA220045772-1a	43311384	6.5G	43076072	6.46G	0.03	96.97	91.77	49.89
VEC_HG1 (HG)	ERRA220045757-1a	43582142	6.54G	43333020	6.5G	0.03	96.67	91.3	49.59
VEC_HG2 (HG)	ERRA220045765-1a	45935402	6.89G	45697224	6.85G	0.03	97.47	92.83	49.84
VEC_HG3 (HG)	ERRA220045773-1a	43745560	6.56G	43494268	6.52G	0.03	96.99	91.84	49.91
VECi_C1 (NGi)	ERRA220045758-1a	52835880	7.93G	52546788	7.88G	0.03	97.34	92.62	50.08
VECi_C2 (NGi)	ERRA220045766-1a	46017410	6.9G	45714780	6.86G	0.03	96.71	91.44	49.66
VECi_C3 (NGi)	ERRA220045774-1a	42451408	6.37G	42237390	6.34G	0.03	97.13	92.13	49.92
VECi_HG1 (HGi)	ERRA220045759-1a	51728474	7.76G	51395098	7.71G	0.03	97.52	93.05	49.9
VECi_HG2 (HGi)	ERRA220045767-1a	40759850	6.11G	40514292	6.08G	0.03	96.46	90.87	49.64
VECi_HG3 (HGi)	ERRA220045775-1a	46606566	6.99G	46340662	6.95G	0.03	97.49	92.94	49.97

Supplementary table S2: Mapping result summary.

sample	total_reads	total_map	Unique map	multi_map	read1_map	read2_map	positive_map	negative_map	splice_map	unsplice_map	proper_map
VEC_C1 (NG)	41349446	39806907(96.27%)	38864378(93.99%)	942529(2.28%)	19492048(47.14%)	19372330(46.85%)	19422461(46.97%)	19441917(47.02%)	15926854(38.52%)	22937524(55.47%)	37965936(91.82%)
VEC_C2 (NG)	37690706	36113871(95.82%)	35325563(93.72%)	788308(2.09%)	17705132(46.97%)	17620431(46.75%)	17645969(46.82%)	17679594(46.91%)	14390482(38.18%)	20935081(55.54%)	34397978(91.26%)
VEC_C3 (NG)	43076072	41024669(95.24%)	39994024(92.85%)	1030645(2.39%)	20029286(46.5%)	19964738(46.35%)	19981640(46.39%)	20012384(46.46%)	16295286(37.83%)	23698738(55.02%)	38901474(90.31%)

VEC_HG1 (HG)	43333020	41321553(95.36%)	40344785(93.1%)	976768(2.25%)	20249512(46.73%)	20095273(46.37%)	20156743(46.52%)	20188042(46.59%)	16198866(37.38%)	24145919(55.72%)	39262178(90.61%)
VEC_HG2 (HG)	45697224	43764145(95.77%)	42665513(93.37%)	1098632(2.4%)	21388052(46.8%)	21277461(46.56%)	21321321(46.66%)	21344192(46.71%)	17108727(37.44%)	25556786(55.93%)	41726918(91.31%)
VEC_HG3 (HG)	43494268	41548139(95.53%)	40527757(93.18%)	1020382(2.35%)	20302290(46.68%)	20225467(46.5%)	20245610(46.55%)	20282147(46.63%)	16318864(37.52%)	24208893(55.66%)	39460948(90.73%)
VECi_C1 (NGi)	52546788	50106166(95.36%)	48784686(92.84%)	1321480(2.51%)	24453672(46.54%)	24331014(46.3%)	24379658(46.4%)	24405028(46.44%)	20032867(38.12%)	28751819(54.72%)	47650926(90.68%)
VECi_C2 (NGi)	45714780	43604319(95.38%)	42528973(93.03%)	1075346(2.35%)	21318788(46.63%)	21210185(46.4%)	21242452(46.47%)	21286521(46.56%)	17127441(37.47%)	25401532(55.57%)	41415670(90.6%)
VECi_C3 (NGi)	42237390	40335700(95.5%)	39327615(93.11%)	1008085(2.39%)	19687912(46.61%)	19639703(46.5%)	19649779(46.52%)	19677836(46.59%)	16003926(37.89%)	23323689(55.22%)	38279938(90.63%)
VECi_HG1 (HGi)	51395098	49418593(96.15%)	48193984(93.77%)	1224609(2.38%)	24136279(46.96%)	24057705(46.81%)	24081687(46.86%)	24112297(46.92%)	19619375(38.17%)	28574609(55.6%)	47086476(91.62%)
VECi_HG2 (HGi)	40514292	38616587(95.32%)	37679978(93.0%)	936609(2.31%)	18935489(46.74%)	18744489(46.27%)	18822067(46.46%)	18857911(46.55%)	15237846(37.61%)	22442132(55.39%)	36645082(90.45%)
VECi_HG3 (HGi)	46340662	44565643(96.17%)	43449666(93.76%)	1115977(2.41%)	21772266(46.98%)	21677400(46.78%)	21707121(46.84%)	21742545(46.92%)	17518791(37.8%)	25930875(55.96%)	42456264(91.62%)

Supplementary Table S3. The sequences of oligonucleotide primers used for evaluation of gene expression.

Gene	Forward primer	Reverse primer
VASP	ATGGCAACAAGCGATGGCT	CGATGGCACAGTTGATGACCA
ROCK1	GGTGGTCGGTTGGGGTATTTT	CGCCCTAACCTCACTTCCC
RhoA	GGAAAGCAGGTAGAGTTGGCT	GGCTGTCGATGGAAAAACACAT
ITGA1	GTGCTTATTGGTTCTCCGTTAGT	CACAAGCCAGAAATCCTCCAT
ITGA3	TGTGGCTTGGAGTGACTGTG	TCATTGCCTCGCACGTAGC
ITGA4	TACAGATGCAGGATCGGAAAGA	AGGTTCTCCATTAGGGCTACC
ITGA5	GCCTGTGGAGTACAAGTCCTT	AATTCGGGTGAAGTTATCTGTGG
ITGAV	GCTGTCGGAGATTTCAATGGT	TCTGCTCGCCAGTAAAATTGT
ITGB1	GTAACCAACCGTAGCAAAGGA	TCCCCTGATCTTAATCGCAAAAC
ITGB2	TTCGGGTCCTTCGTGGACA	ACTGGTTGGAGTTGTTGGTCA
ITGB3	CATGAAGGATGATCTGTGGAGC	AATCCGCAGGTTACTGGTGAG
ITGB5	GGAAGTTCGGAAACAGAGGGT	CTTTCGCCAGCCAATCTTCTC
FAK	AGTGGACCAGGAAATTGCTTTG	GTGTTTTGGCCTTGACAGAATC
CAV1	CATCCCGATGGCACTCATCTG	TGCACTGAATCTCAATCAGGAAG
JAM2	AACTGGGTCGGAGTGTCTC	GGGGCACTAACTTCACAACGA
CLDN5	CTCTGCTGGTTCGCCAACAT	CAGCTCGTACTTCTGCGACA
CDH2	TGCGGTACAGTGTAACTGGG	GAAACCGGGCTATCTGCTCG
CDH5	TTGGAACCAGATGCACATTGAT	TCTTGCGACTCACGCTTGAC
CDH11	CCATCCCAAGAGAGGTCTGC	TAGGCACAGAATGCAGC
ICAM1	TTGGGCATAGAGACCCCGTT	GCACATTGCTCAGTTCATACACC
VCAM1	TTGGGCATAGAGACCCCGTT	GCACATTGCTCAGTTCATACACC
PECAM1	CCAAGGTGGGATCGTGAGG	TCGGAAGGATAAAACGCGGTC
SELE	GGGAAGATGGTCGTGATCCTT	TCTGGGGTGGTCTCGATTTTA
VWF	CCTTGACCTCGGACCCTTATG	GATGCCCGTTCACACCACT

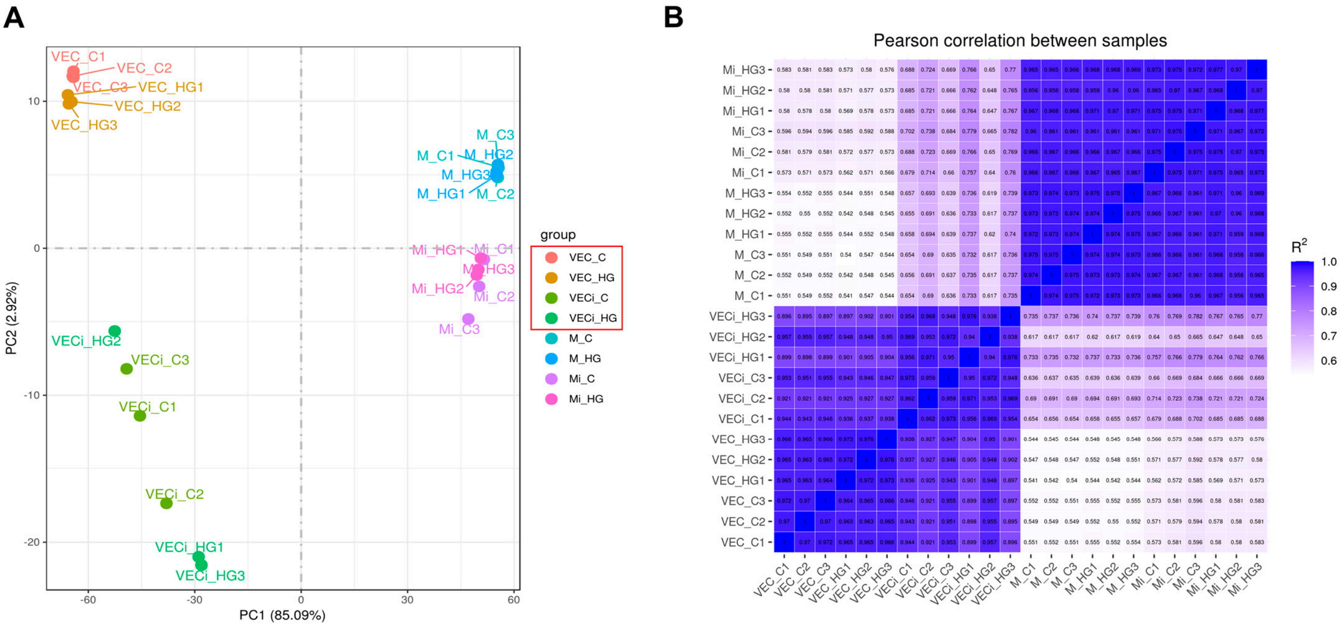

Supplementary Figure S1: PCA and Pearson correlation coefficient matrix. The Pearson correlation matrix show a high correlation between replicates. VEC_C represents VECs in normal glucose (NG), VEC_HG represents VECs in high glucose (HG), VEC_Ci represents VECs interacted with monocytes in normal glucose (NGi) and VECi_HG represents VECs interacted with monocytes in high glucose (HGi).

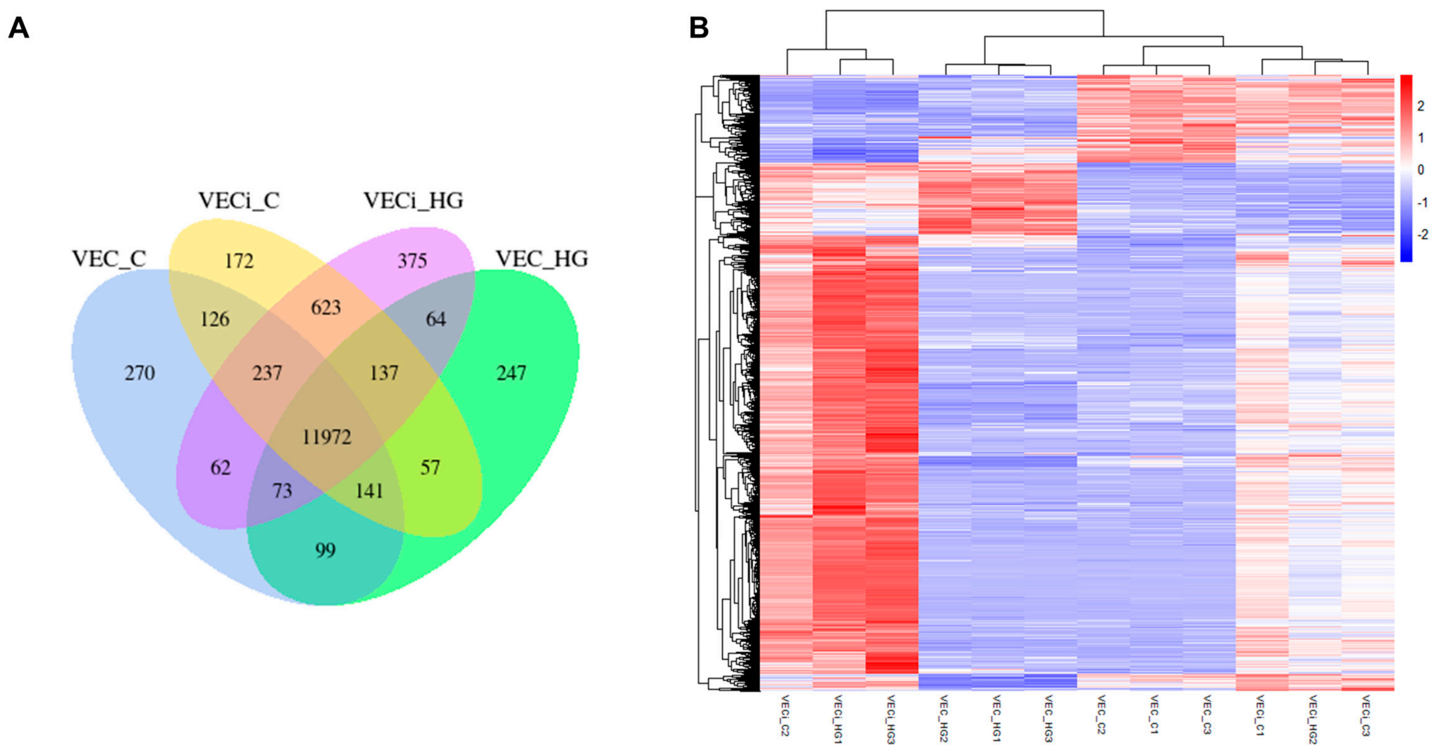

Supplementary Figure S2: VENN diagram of all samples. Heatmap of all genes with fold change >1.5 and padj<0.05. VEC_C represents VECs in normal glucose (NG), VEC_HG represents VECs in high glucose (HG), VEC_Ci represents VECs interacted with monocytes in normal glucose (NGi) and VECi_HG represents VECs interacted with monocytes in high glucose (HGi).

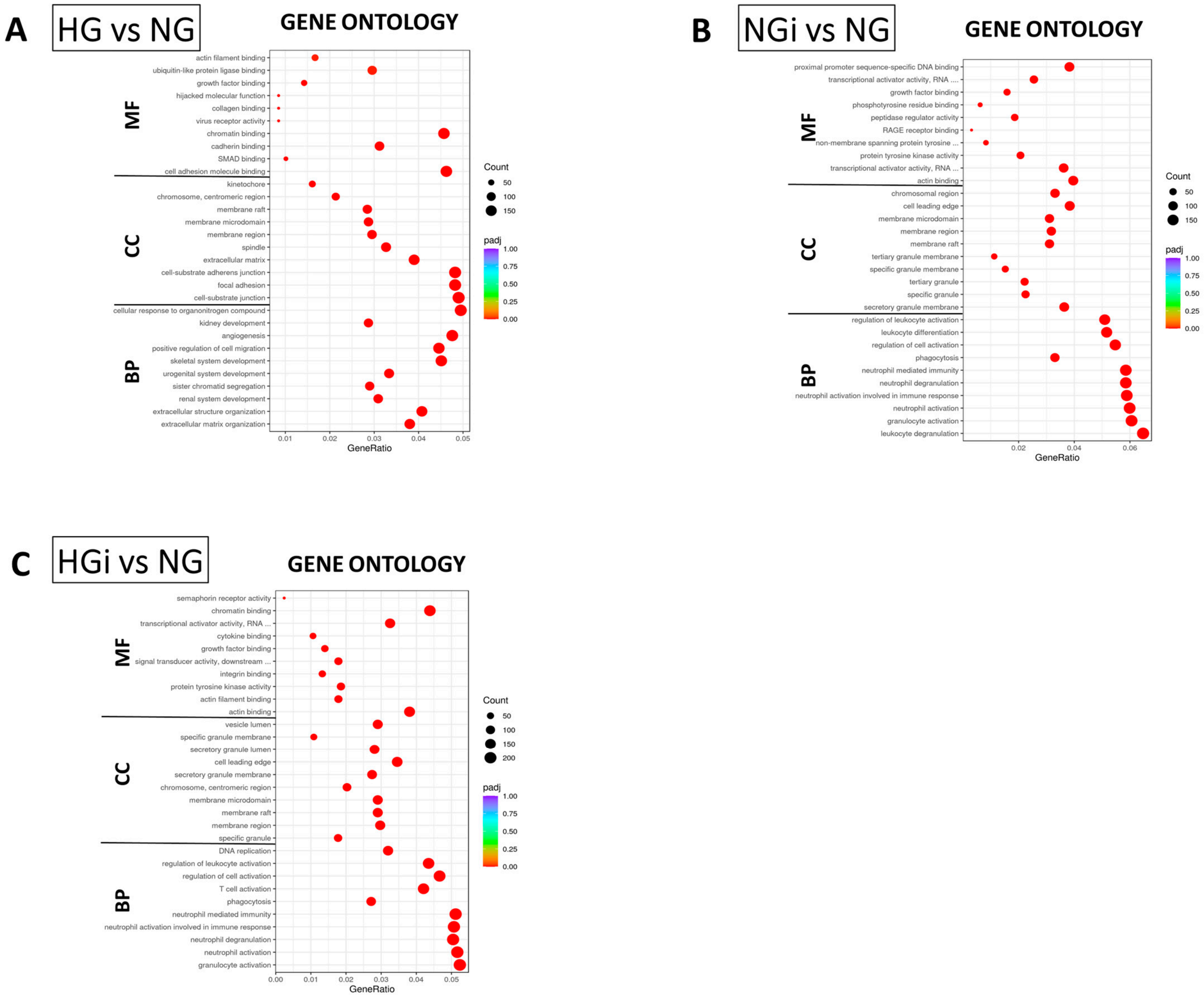

Supplementary Figure S3. Gene ontology analysis of DEGs in HG, NGi and HGi compared to NG. The top 10 enrichements in Molecular function (MF), Cellular compartment (CC) and Biological process (BP) are depicted for each comparison.
